# Prognostic Significance of Bcl-2 Expression in Non-germinal Center B-cell-Like Diffuse Large B-cell Lymphoma

**DOI:** 10.7759/cureus.62031

**Published:** 2024-06-09

**Authors:** Dylan Alford, Ivan Petković

**Affiliations:** 1 Clinical Oncology, University of Niš, Niš, SRB

**Keywords:** diffuse large b-cell lymphoma, rituximab, bcl-2, non-gcb, non-hodgkin lymphoma, dlbcl

## Abstract

Introduction: Diffuse large B-cell lymphomas (DLBCLs) are a group of malignant neoplasms with extensive clinical and molecular heterogeneity. Several key genetic aberrations have been identified, such as those involving the *MYC*, *BCL6*, and *BCL2* genes. Prior studies on the prognostic significance of Bcl-2 protein expression in DLBCL have been contradictory, with some suggesting it has an adverse effect, while others have shown no such association. Bcl-2 is known to be more highly expressed in the non-germinal center B-cell-like (non-GCB) subtype compared to germinal center B-cell-like (GCB) DLBCL. Non-GCB status is associated with a less favorable prognosis. This study aimed to investigate whether the expression of Bcl-2 protein in non-GCB DLBCL influences response to treatment, progression-free survival, or overall survival.

Methods: In this retrospective study, we investigated whether there was a difference in the clinical outcomes of non-GCB DLBCL cases (n = 97) that were confirmed by immunochemistry to demonstrate high levels of Bcl-2 protein expression (>50% neoplastic cells stained) when compared to those who were deemed negative based on this criterion. Response to rituximab-based induction immunochemotherapy, five-year progression-free survival, and five-year overall survival were assessed.

Results: There was no statistically significant difference in response to treatment, five-year progression-free survival, or five-year overall survival between the patients who were positive for Bcl-2 (n = 70) compared to those who were considered Bcl-2 negative (n = 27).

Conclusion: High levels of Bcl-2 protein expression do not appear to be of prognostic significance in non-GCB DLBCL and therefore Bcl-2 may not be a key therapeutic target in the treatment and improvement of clinical outcome in such cases.

## Introduction

Non-Hodgkin's lymphomas (NHLs) are a heterogeneous group of malignant neoplasms originating from lymphoid tissues, the most common of which is diffuse large B-cell lymphoma (DLBCL), accounting for 25-35% of cases in developed countries [1]. DLBCL usually presents with a rapidly enlarging symptomatic mass, typically a nodal enlargement in the neck or abdomen, but a mass lesion can also arise in extranodal tissues. Rituximab is a chimeric monoclonal antibody targeted against CD20 B-cell surface antigens that has been proven to be an integral component of immunochemotherapy regimens for the treatment of DLBCL since its introduction.

Two molecular subtypes of DLBCL have been defined by the World Health Organization: germinal center B-cell (GCB) and the non-GCB/activated B-cell (ABC) subtype [1]. Gene expression profiling and immunohistochemistry are techniques used to determine the cell of origin once a surgical excision biopsy or fine-needle aspirate has been performed.

The Hans algorithm has been proposed as a means by which GCB and non-GCB subtypes of DLBCL can be identified by immunohistochemistry. This is particularly useful in countries where gene expression profiling is not a widely available technique. The Hans method uses CD10, BCL6, and MUM1 as markers. Samples expressing CD10 are classified as GCB and those that are negative for CD10 are tested for BCL6 and are classified as non-GCB if negative. If the sample is CD10 negative and BCL6 positive, then MUM1 expression is evaluated. If positive for MUM1, the cases are classified as non-GCB, or they are classified as GCB if negative for MUM1 [2].

Common cytogenetic abnormalities in DLBCL are known to involve the *MYC*, *BCL6*, and *BCL2 *genes. Of particular interest right now is the *BCL2* gene located at 18q21.33. Its product, Bcl-2 (B-cell lymphoma 2), is localized to the outer mitochondrial membrane where it acts to inhibit pro-apoptotic proteins belonging to the same Bcl-2 family of regulator proteins, namely, Bax and Bak, which would otherwise promote the release of cytochrome c and reactive oxygen species from mitochondria, inducing apoptosis [3]. Bcl-2 is known to be more highly expressed in non-GCB DLBCL and we know that this subtype is associated with poorer clinical outcomes when compared to GCB DLBCL [4,5]. Despite this, the prognostic significance of Bcl-2 protein expression remains a point of controversy within this area of hemato-oncology, necessitating further research.

In this study, we sought to elucidate the prognostic significance of Bcl-2 protein expression in non-GCB DLBCL. Our main objectives were to (i) assess the effect of Bcl-2 protein expression on both overall and progression-free survival in non-GCB DLBCL patients treated with rituximab-based immunochemotherapy, and (ii) identify any differences in the response to treatment among those highly expressing Bcl-2 when compared to those who do not.

## Materials and methods

Patient inclusion criteria

We included all patients diagnosed and treated by the Clinic of Oncology at the University Clinical Centre in Niš, Serbia who met the following criteria: (i) diagnosis of DLBCL confirmed by histopathological analysis; (ii) treated by rituximab-based immunochemotherapy; (iii) negative for human immunodeficiency virus; (iv) did not die of unrelated causes or with COVID-19 infection; (v) treated at the clinic between 2008 and the end of 2023.

Immunohistochemical analysis

The tissue samples analyzed for our study were collected from patients with suspected DLBCL for routine diagnostic purposes. All immunohistochemical staining was performed at the relevant Serbian reference institutes located in the cities of Belgrade, Niš, and Novi Sad. Biopsy material is delivered to the reference institutes in 10% buffered formalin, sectioned, and then fixed in 10% buffered formalin for 24 hours before being molded in liquid paraffin. Tissue microarrays are then prepared from the paraffin-embedded diagnostic biopsy specimens. Five-micrometer sections are cut from each tissue microarray and stained with monoclonal antibodies for Bcl-2 (clone 124, Dako, Glostrup, Denmark), BCL6 (clone PG-B6p, Dako), CD10 (clone 56C6, Dako), and MUM1 (Dako). Cases are identified as being GCB or non-GCB according to the Hans algorithm using 30% cut-offs for CD10, BCL6, and MUM1 protein expression. Cases are considered positive for Bcl-2 if greater than 50% of neoplastic cells were stained by the antibody.

International prognostic index

At the time of diagnosis, the performance status of patients was assessed according to the Eastern Cooperative Oncology Group (ECOG) criteria, their cancer was staged according to the Ann Arbor system, and a serum lactate dehydrogenase (LDH) level was recorded. Using these data, we calculated the International Prognostic Index (IPI) scores for patients over the age of 60 and age-adjusted IPI scores for those who were younger than 60 years old.

Bulky disease and bone marrow involvement

Computed tomography (CT) was used to identify whether a patient had bulky disease, which was defined as the presence of a single nodal mass of ≥7 cm in maximum dimension. Bone marrow involvement was assessed by iliac crest bone marrow biopsy at the time of diagnosis.

Data collection

According to the inclusion criteria, a total of 97 patients were identified, 68 of whom had been followed up at the clinic for a period of at least five years, and their data were used to analyze five-year progression-free and five-year overall survival (51 Bcl-2 positive and 17 Bcl-2 negative). Statistical analysis of response to induction treatment and both progression-free and overall survival throughout the duration of our study involved all 97 patients identified by the inclusion criteria. At the clinic, patients with a confirmed diagnosis of DLBCL are followed up at three-month intervals after receiving their final cycle of rituximab-based induction therapy for the first two years, then at six-month intervals for the next three years, and then on a yearly basis. Medical records were checked retrospectively to identify whether any of the patients involved in the study had been deemed to have progressive disease or were deceased as of the end of 2023. If so, the date on which they had died, or their disease had progressed was recorded for Kaplan-Meier survival analysis. Complete response was defined as the complete disappearance of radiologic evidence of disease on CT scan; partial response as a 50% or greater decrease in the product of perpendicular diameters, or their sum if multiple lesions were present; progressive disease as the appearance of new lesions or recurrence of previously resolved lesions. Response to treatment was examined retrospectively using the medical documentation of patients who had met the inclusion criteria for our study.

Statistical analysis

Group comparisons in our study were performed using Student’s t-test and the chi-squared test, depending on the type of variable in question. Time-to-event data were analyzed using IBM SPSS Statistics version 26 (IBM Corp., Armonk, NY) to produce Kaplan-Meier survival estimates. Overall survival was defined as the time from the date of diagnosis to death associated with a diagnosis of DLBCL. Progression-free survival was the time from the initial diagnosis to the date of cancer progression or death. Obtained p-values < 0.05 were considered statistically significant.

## Results

Patient characteristics

A total of 97 patients with non-GCB DLBCL who met all the inclusion criteria were identified. Immunohistochemical analysis determined that 70 of the 97 cases were positive for Bcl-2 protein expression (72.2%). The clinical characteristics of these patients at the time of diagnosis, divided according to Bcl-2 protein expression status, are listed in Table 1. No significant differences were identified in the baseline clinical characteristics of the two groups.

**Table 1:** Clinical characteristics of the 97 DLBCL patients involved in our study, separated according to Bcl-2 protein expression status. DLBCL: diffuse large B-cell lymphoma; IPI: International Prognostic Index.

Characteristics	Bcl-2 positive (n = 70)	Bcl-2 negative (n = 27)	p-value
Mean age in years (range, SD)	60.0 (23-85, 15.6)	58.0 (29-78, 13.9)	0.28
Sex (%)			0.35
Male	41	52	
Female	59	48	
Mass size (%)			0.60
<7 cm	54	63	
>7 cm	46	37	
Lactate dehydrogenase (%)			0.14
Normal	50	67	
Elevated	50	33	
Site of presentation (%)			0.63
Primary nodal	39	33	
Primary extranodal	61	67	
Ann Arbor stage (%)			0.97
I-II	49	44	
III-IV	51	56	
IPI score (%)			0.84
0-1	39	41	
>2	61	59	

The highest proportion of elevated LDH levels at diagnosis was found among the Bcl-2-positive group (50%), compared to the 33% for Bcl-2-negative patients. Of the 97 patients involved in our study, 82 were confirmed to be DLBCL, not otherwise specified (NOS); one patient had T-cell-rich large B-cell lymphoma; two cases had primary central nervous system DLBCL; 12 patients had primary mediastinal large B-cell lymphoma.

Response to rituximab-based immunochemotherapy

**Figure 1:**
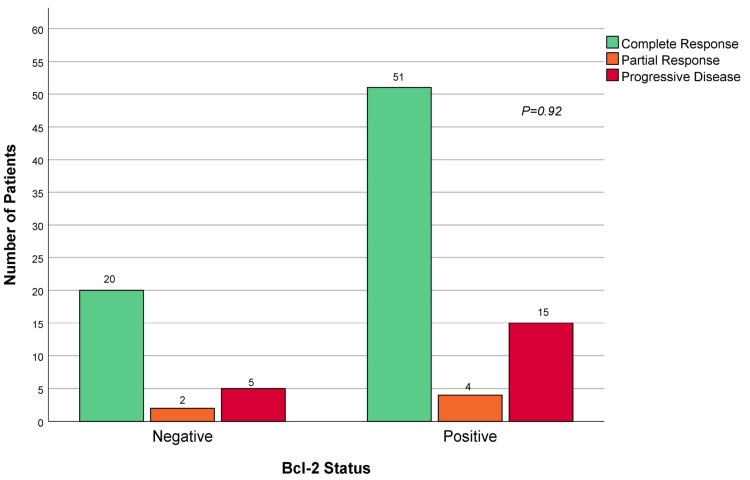
Response to rituximab-based induction immunochemotherapy according to Bcl-2 protein expression status.

Of the 97 patients involved in our study, 76 received at least three cycles of R-CHOP (rituximab, cyclophosphamide, doxorubicin, vincristine, and prednisolone) (78.4%), eight received at least six cycles of DA-EPOCH-R (dose-adjusted etoposide, prednisone, vincristine, cyclophosphamide, doxorubicin, and rituximab) (8.2%), 10 received at least three cycles of R-CVP (rituximab, cyclophosphamide, vincristine, and prednisolone) (10.3%), one received three cycles of R-CHOEP (rituximab, cyclophosphamide, doxorubicin, vincristine, etoposide, and prednisolone) (1.0%), and two patients received at least six cycles of R-HDMTX (rituximab with high-dose methotrexate) (2.1%). The response of these patients to these rituximab-based regimens is presented in Figure 1. Complete response was achieved in 74.1% of Bcl-2-negative patients and in 72.9% of Bcl-2-positive patients; partial response was achieved in 7.4% of Bcl-2-negative cases and in 5.7% of Bcl-2-positive cases; progressive disease was identified following induction therapy in 18.5% of Bcl-2-negative cases versus 21.4% of Bcl-2-positive cases. No significant differences in response were identified between the two groups (p = 0.92).

Prognostic significance of Bcl-2 protein expression

**Figure 2:**
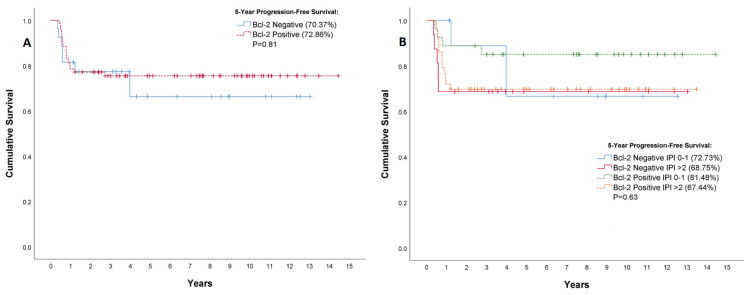
Correlation between Bcl-2 protein expression and progression-free survival among patients with non-germinal center B-cell (non-GCB) diffuse large B-cell lymphoma. Survival is shown for all patients combined (A), or according to their International Prognostic Index score at diagnosis (B). Vertical tick marks indicate censored data.

When calculating five-year progression-free survival, which considers time from diagnosis until progression of the disease or death, no statistically significant difference was identified between the Bcl-2-negative and Bcl-2-positive patient groups (p = 0.81) (Figure 2A). When separating the cohort according to IPI score at diagnosis (Figure 2B), the lowest progression-free survival rates were again seen at IPI scores >2 with values of 67.44% and 68.75% for Bcl-2-positive and negative cases, respectively, although this difference was not statistically significant (p = 0.63).

Mantel-Cox test analyses were conducted on the progression-free survival distributions of the entire cohort spanning the full duration of our study, according to Bcl-2 protein expression status (p = 0.56) and when grouped according to IPI score (p = 0.43), with no significant difference in progression-free survival identified between the groups.

**Figure 3:**
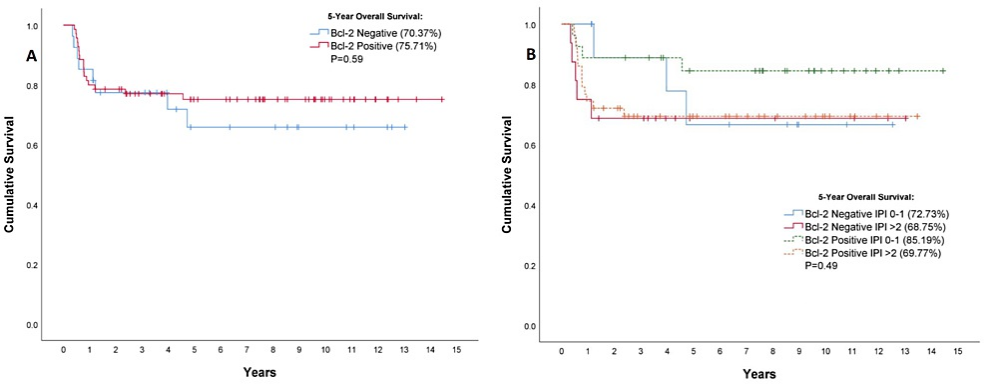
Correlation between Bcl-2 protein expression and overall survival among patients with non-germinal center B-cell (non-GCB) diffuse large B-cell lymphoma. Survival is shown for all patients combined (A), or according to their International Prognostic Index score at diagnosis (B). Vertical tick marks indicate censored data.

In terms of five-year overall survival, there was no significant difference between the Bcl-2-negative and Bcl-2-positive patient groups when analyzing the entire cohort (p = 0.59) (Figure 3A). Subgroup analysis of the overall survival was performed by dividing the patients according to their IPI score at diagnosis (Figure 3B). The lowest rates of five-year overall survival were seen among Bcl-2-positive patients (69.8%) and negative patients (68.8%) with IPI scores greater than 2, although this was not of statistical significance (p = 0.49).

A Mantel-Cox test was conducted on the survival distributions of the entire cohort spanning the full duration of the study (2008-2023), according to Bcl-2 protein expression status (p = 0.55) and when grouped according to IPI score (p = 0.42), with no significant difference in overall survival observed between the groups.

## Discussion

Bcl-2 is an anti-apoptotic protein, encoded by the *BCL2* gene, belonging to the Bcl-2 family of regulator proteins. It is known that Bcl-2 expression provides a resistance mechanism to genotoxic insults, such as radiation or chemotherapy [6]. In this study, we investigated the prognostic significance of Bcl-2 protein expression on response to existing rituximab-based immunochemotherapy regimens and its influence on both five-year overall and progression-free survival in non-GCB DLBCL, of which it was highly expressed in 72.2% of cases (Table 1). The proportion of Bcl-2 positive cases is consistent with reports that two-thirds of non-GCB DLBCL patients will demonstrate high levels of expression [7], as are the mean patient ages at diagnosis because DLBCL is usually diagnosed in the sixth decade of life.

When assessing whether there was a relationship between Bcl-2 expression status and response to current induction therapy regimens, we found that although a greater proportion of Bcl-2-negative patients achieved complete or partial response compared to those who highly expressed Bcl-2, this difference was not significant. It was also noted that a higher proportion of Bcl-2-positive cases were found to have progressive disease at follow-up, but again this did not reach statistical significance (Figure 1).

We then sought to evaluate both the overall and progression-free survival of the cohort. We did not identify an association between high levels of Bcl-2 protein expression (>50% of neoplastic cells expressing Bcl-2 protein) and inferior five-year or longer-term survival. Existing markers that are broadly implicated in an unfavorable prognosis include elevated LDH levels, increasing IPI scores, and higher Ann Arbor stages. The highest proportion of cases with elevated LDH at diagnosis was recorded in the Bcl-2-positive group (50%) compared to the 33% of those cases whose Bcl-2 status was considered negative, but this did not appear to translate to poorer clinical outcomes. Ann Arbor staging and calculated IPI scores were comparable across both groups (Table 1). Greater numbers of bulky disease cases were seen amongst those highly expressing Bcl-2, but in the rituximab era of DLBCL treatment, this is regarded as a less consequential prognostic factor [8].

There are many contradictory reports in the literature about the prognostic impact of Bcl-2 protein expression. For example, Iqbal et al. also concluded that non-GCB patients treated with rituximab-containing regimens did not demonstrate poorer clinical outcomes [9], but other groups have suggested that Bcl-2 is an unfavorable prognostic indicator of overall survival [10]. Of interest, it has been noted previously that Bcl-2-positive non-GCB DLBCL tumors may benefit proportionally more from the addition of rituximab to CHOP (cyclophosphamide, doxorubicin, vincristine, and prednisolone) regimens than Bcl-2-negative non-GCB tumors [9], narrowing differences in survival, which may explain this finding in our study. Without rituximab, Bcl-2-positive non-GCB DLBCL has been associated with a poorer outcome than highly expressive GCB variants [9].

There is a consensus that the prognosis is less favorable in non-GCB DLBCL when compared to GCB DLBCL. It is possible that prognostic markers other than Bcl-2 may have a more pronounced impact on survival. For instance, we know that in non-GCB DLBCL there is high expression of target genes of the nuclear factor (NF)-κB transcription factors, with activity of the NF-κB pathway being implicated in the poor prognosis of these patients [11]. This pathway can be activated by B cell receptor (BCR) survival signaling components, such as Bruton’s tyrosine kinase (Btk). Pharmacological inhibition of Btk using ibrutinib has been shown to selectively kill non-GCB DLBCL cells [12].

In terms of targeted pharmacological therapy, there is a current research focus on the use of venetoclax, a Bcl-2 homology domain 3 (BH3) mimetic. By binding to Bcl-2 and blocking its actions, it has proven efficacious for the treatment of other cancers where the Bcl-2 protein is highly expressed, such as chronic lymphocytic leukemia. Therefore, it will be of interest to identify whether Bcl-2-positive DLBCL tumors are more responsive to a combination of venetoclax and R-CHOP than R-CHOP alone. Clinical trials such as the CAVALLI study are currently investigating this [13].

Limitations

The use of immunohistochemistry and the Hans algorithm to assign cases to the GCB or non-GCB subtype is not without its flaws and may explain, at least in part, the findings of our research. When compared to gene expression profiling, the Hans algorithm has demonstrated a sensitivity of 85-90% and a specificity of 52-82% in determining cell of origin [14]. In a study by Read et al., non-GCB patients fared significantly worse than those with GCB DLBCL when they were assigned a subtype by gene expression profiling [5]. When they were designated according to the Hans immunohistochemistry algorithm, no difference was found between the two subtypes in terms of overall survival [5]. Therefore, it is possible that the outcome of our study may have been different had gene expression profiling been used to make this distinction.

## Conclusions

In summary, no statistically significant differences were identified between the two patient groups in response to treatment, five-year progression-free survival, or five-year overall survival. Therefore, we can conclude that high levels of Bcl-2 expression do not appear to be of prognostic significance among non-GCB DLCBCL cases that have been designated to this subtype by use of the Hans immunohistochemistry algorithm. This necessitates further research to identify whether an association may be present when gene expression profiling is used and whether drugs such as venetoclax could improve clinical outcomes for patients with non-GCB DLBCL.
